# Unveiling Insight: Autopsy findings on Alzheimer’s Disease Clinical Trial Participants and the multifaceted existence of comorbid pathologies

**DOI:** 10.1002/alz.090032

**Published:** 2025-01-09

**Authors:** Elif PINAR Coskun, Justin M Barber, Erin L. Abner, Frederick A Schmitt, Larry B Goldstein, Linda J. J. Van Eldik, Peter T Nelson, Gregory A Jicha

**Affiliations:** ^1^ University of Kentucky, SBCoA, Lexington, KY USA; ^2^ University of Kentucky College of Medicine, Lexington, KY USA; ^3^ Department of Epidemiology, University of Kentucky, Lexington, KY USA; ^4^ Sanders‐Brown Center on Aging, University of Kentucky, Lexington, KY USA; ^5^ Sanders‐Brown Center on Aging, Lexington, KY USA; ^6^ University of Kentucky, Lexington, KY USA; ^7^ University of Kentucky/Sanders‐Brown Center on Aging, Lexington, KY USA; ^8^ University of Kentucky Sanders‐Brown Center on Aging, Lexington, KY USA

## Abstract

**Background:**

The presence of multiple comorbid pathologic features in late‐onset dementia has been well documented across cohort studies that incorporate autopsy evaluation. It is likely that such mixed pathology potentially confounds the results of interventional trials that are designed to target a solitary pathophysiologic mechanism in Alzheimer’s disease and related dementias (ADRD).

**Method:**

The UK ADRC autopsy database was screened for participants who had previously engaged in therapeutic interventional trials for Alzheimer’s disease, vascular cognitive impairment, dementia, and/or ADRD prevention trials from 2005 to the present. Sixty‐five cases (out of a total n = 534) were engaged in clinical trials for the prevention or treatment of ADRD. Pathologic features studied included b‐amyloid, tau, a‐synuclein, TDP‐43, and cerebrovascular disease using conventional rating scales.

**Result:**

Autopsy cases for those who previously engaged in clinical trials did not differ significantly from those that were trial‐naïve for demographic, genetic (ApoE), or clinical characteristics except for CDR global scores that were lower for past trial participants (p<0.05). Comorbid pathologies were common across study types with a mean of 3 pathologic features/participant, 17% with quadruple misfolded proteinopathy (QMP; Ab+tau+synuclein+ TDP43). Yet only 10% of MCI/AD trial participants demonstrated a “pure” disease state that was targeted by the study intervention.

**Conclusion:**

In our study, approximately 2 out of 3 ADRD trial participants had comorbid mixed pathologic features. Understanding the heterogeneity of pathologies seen in clinical trial participants may allow improved inclusion/exclusion criteria based on clinical features and the rational use of antemortem biomarkers to stratify the likelihood of mixed comorbid pathology. In addition, the recognition will allow the usage of combination therapy for future interventional strategies. Such insights may also enable improved power analyses and statistical designs that may be able to adjust for the confounds of such mixed pathologic disease states that are common in ADRD clinical trials.

